# Role of pulmonary rehabilitation in extracellular matrix protein expression in vastus lateralis muscle in atrophic and nonatrophic patients with COPD

**DOI:** 10.1183/23120541.00543-2024

**Published:** 2025-01-20

**Authors:** Efpraxia Kritikaki, Gerasimos Terzis, Meera Soundararajan, Ioannis Vogiatzis, Davina C.M. Simoes

**Affiliations:** 1 Faculty of Health and Life Sciences, Northumbria University Newcastle, Newcastle upon Tyne, UK; 2 Sports Performance Laboratory, School of Physical Education and Sports Science, National and Kapodistrian University of Athens, Athens, Greece

## Abstract

**Background:**

In response to exercise-based pulmonary rehabilitation (PR), the type of muscle fibre remodelling differs between COPD patients with peripheral muscle wasting (atrophic patients with COPD) and those without wasting (nonatrophic patients with COPD). Extracellular matrix (ECM) proteins are major constituents of the cell micro-environment steering cell behaviour and regeneration. We investigated whether the composition of ECM in atrophic compared to nonatrophic patients with COPD differs in response to PR.

**Methods:**

Vastus lateralis muscle biopsies from 29 male COPD patients (mean±sem forced expiratory volume in 1 s: 43±6% predicted) classified according to their fat-free mass index as atrophic (<17 kg·m^−2^, n=10) or nonatrophic (≥17 kg·m^−2^, n=19) were analysed before and after a 10-week PR programme for myofibre distribution and size, whereas a selection of ECM molecules was quantified using ELISA and real-time PCR.

**Results:**

In nonatrophic patients with COPD PR was associated with increased myofibre type I distribution (by 6.6±2.3%) and cross-sectional area (CSA) (by 16.4±4.8%), whereas in atrophic patients with COPD, PR induced increased myofibre type IIa distribution (by 9.6±2.8%) and CSA (by 12.1±3.2%). PR induced diverse intramuscular ECM adaptations in atrophic compared to nonatrophic patients with COPD. Accordingly, following PR there was a significant increase in protein levels of ECM biomarkers (collagen type I by 90 pg·mL^−1^; collagen type IV by 120 pg·mL^−1^; decorin by 70 pg·mL^−1^) only in nonatrophic patients with COPD. Conversely, post-PR, osteopontin, a protein known for its dystrophic effects, and tenacin C, a necroptosis compensatory factor facilitating muscle regeneration, were upregulated at protein levels (by 280 pg·mL^−1^and 40 pg·mL^−1^, respectively) in atrophic patients with COPD, whereas fibronectin protein levels were decreased.

**Conclusions:**

These findings suggest that the differential PR-induced myofibre adaptations in atrophic compared to nonatrophic patients with COPD could be associated with inadequate remodelling of the intramuscular ECM environment.

## Shareable abstract

**The diverse ECM protein adaptation in response to PR in atrophic compared to nonatrophic patients with COPD highlights novel mechanisms for myofibre atrophy. Identification of atrophy-specific ECM proteins could offer new therapeutic prospects.** https://bit.ly/4ckPono

## Introduction

The limb muscles in patients with COPD are atrophied, weak, fatigable and metabolically inefficient [1]. Multiple aetiologies underlie the loss of contractile myofibre including disuse, oxidative stress, local inflammation, hypoxaemia, protein anabolism/catabolism imbalance and impaired regenerative capacity [1]. These unfavourable muscle characteristics concur to limit exercise capacity, a most debilitating feature in these patients [2]. Exercise-based pulmonary rehabilitation (PR) increases muscle mass and myofibre cross-sectional area (CSA) in tandem with protein accretion superseding catabolism [3]. COPD patients with peripheral muscle wasting (atrophic patients with COPD) partially retain the capacity for peripheral muscle remodelling in response to PR and can increase exercise capacity as much as nonatrophic patients with COPD, even if they exhibit both quantitative and qualitative differences in the type of muscle fibre remodelling in response to PR [4]. However, it remains poorly understood why the peripheral muscle myofibres in atrophic patients with COPD can only partially remodel in response to PR.

Alongside the decrease in myofibre size, the non-contractile extracellular matrix (ECM) is proportionally increased in ageing and muscle unloading [5]. The ECM is a complex network of collagens, proteoglycans and glycoproteins forming the cell's micro-environment. Besides providing structural support and force transmission, the ECM also serves as a mechanical signal transducer that steers cell behaviour and muscle adaptation. It acts as a reservoir for growth factors and creates concentration gradients that can directly impact myofibre hypertrophy [6]*.* Age-dependent alterations in ECM composition contribute to impaired muscle repair and reduced force production [6, 7]. Several myopathies result from gene mutations that disturb ECM components, including various collagens, tenascin and osteopontin [8]. In COPD and ageing, ECM remodelling is among the most affected signalling pathways in peripheral muscles [9, 10].

Our recent work indicates that despite the decreased contractile muscle tissue in atrophic patients with COPD, the differences in ECM mRNA expression for collagen, fibronectin, tenascin C and biglycan between atrophic and nonatrophic patients with COPD are not translated at the protein level [11]. This could potentially indicate an accumulation of long-lived ECM proteins and dysregulated proteostasis that is often observed during deconditioning and ageing [12].

A growing body of evidence demonstrates that in ageing, altered intramuscular ECM composition profoundly hinders the capacity for muscle adaptation in response to exercise training [9]. Likewise, myofibre mechano-transduction occurs when mechanical perturbations of the ECM composition activate cell transmembrane integrins, catalysing downstream signalling to induce protein synthesis [13]. Integrins are transmembrane linkers (integrators) mediating the interactions between the cytoskeleton and the ECM that are required for cells to adhere to the matrix [13]. In addition, the microdamage to myofibres observed after exercise triggers a repair adaptive hypertrophic response [14]. Thus, the precise regulation of muscle ECM production is necessary during periods of adaptation to ensure adequate muscle function.

Whether differences in the ECM profile between atrophic and nonatrophic patients with COPD can be modified in response to a PR programme currently remains unexplored [11]. This retrospective analysis aims to provide novel insights into the biological mechanisms of PR-induced myofibre adaptation by documenting the effects of exercise-based PR on intramuscular ECM composition at both transcription and translation levels in atrophic and nonatrophic patients with COPD. Here we report a set of ECM proteins including collagens that are responsible for maintaining tissue structure (collagen I and IV), proteoglycans (decorin and biglycan) that are known to enable tissue to withstand compression forces, and matricellular proteins (tenacin, SPARC and osteopontin) which regulate cell response [6]. Understanding how mechanical loading affects ECM composition in atrophic COPD is essential to accelerate the development of pharmacological and non-pharmacological interventions.

## Material and methods

### Study population

We analysed *vastus lateralis* muscle specimens from 29 clinically stable, male COPD patients [4]. All patients met the following entry criteria: 1) post-bronchodilator forced expiratory volume in 1 s (FEV_1_) <50% predicted and FEV_1_/forced vital capacity (FVC) <0.70 without significant post-bronchodilator reversibility; and 2) optimal medical therapy without regular use of systemic corticosteroids. Fat-free mass index (FFMI) was assessed by bioelectrical impedance (Bodystat 1500; Bodysat Ltd, Isle of Man, UK) to classify COPD patients as atrophic (FFMI <17 kg·m^−2^) or nonatrophic (FFMI ≥17 kg·m^−2^) [15]. Muscle specimens were stored and analysed at Northumbria University, Newcastle in accordance with the Human Tissue Act 2004 and with approval from Northumbria University, Newcastle Ethics Committee (HLSIV220916-V2, Newcastle upon Tyne, UK).

### Study design

Patients had completed a 10-week multidisciplinary PR programme as previously described [4]. The programme comprised supervised exercise training (including both interval aerobic and resistance exercise), breathing control and relaxation techniques, methods of clearance of pulmonary secretions, disease education, dietary advice and psychological support on issues relating to chronic disability. Percutaneous biopsies of the right vastus lateralis muscle were performed at mid-thigh (15 cm above the patella) by the Bergstrom technique before (pre-PR) and upon completion of an exercise-based PR programme (post-PR) as described elsewhere [4]. Muscle biopsies were frozen in liquid nitrogen immediately after excision and maintained at −80 °C until analysis for fibre type classification, and fibre CSA [4]. A selection of ECM biomarkers was studied based on their role on myogenesis as previously described [9, 11].

### Quantitative real-time PCR analysis

Total RNA was extracted and reverse transcribed into cDNA and quantified using the quantitative real-time PCR procedure as previously described [11, 16]. Primers used in this study are as detailed elsewhere [11, 16]. Quantitative real-time PCR data are presented as fold changes relative to the housekeeping gene glyceraldehyde-3-phosphate dehydrogenase (GAPDH), estimated using the 2^−ΔΔCt^ method. Gene expression of the following ECM molecules was analysed: collagen type I heterodimer chains (*COL1A1* and *COL1A2*), collagen IV (*COL4A1*), fibronectin (*FN1)*, tenascin C (*TNC*), integrin β1 (*ITGB1*), osteopontin (*SPP1*), secreted protein acidic and rich in cysteine (*SPARC*), decorin (*DCN*) and biglycan (*BGN*). To gain insight as to whether the changes in ECM in COPD muscle are indicative of their myogenic potential, the expression of the myogenic regulatory factors (MRFs) paired box 7 (*PAX7*) was examined [17].

### Protein extraction and analyses

Total protein extraction and analyses were as previously described [11, 16]. Total protein loading onto ELISA was normalised as 1 μg to quantify collagen I (ab285250, Abcam); collagen IV (orb562147) from Biorbyt; osteopontin (DOST00), fibronectin (DFBN10) and SPARC (DSP00) from R&D Systems; tenascin C (EH446RB), decorin (EHDCN) and biglycan (EH45RB) from Invitrogen according to the manufacturer's instructions. ELISA sensitivity and detection range have been described elsewhere [11].

### Statistical analyses

Data on participant demographics and muscle fibre morphology are presented as mean±sem. sem was chosen rather than sd because we were interested in the variance of the mean values rather than the inter-subject variance. Baseline demographic and muscle fibre morphological characteristics between nonatrophic and atrophic patients with COPD were compared by unpaired t-tests*.* Differences within atrophic or nonatrophic patients with COPD pre- and post-PR were analysed by the Wilcoxon matched pairs signed rank test. Between COPD group differences following completion of PR were analysed using an unpaired two-tailed Mann–Whitney test, and data are presented as median (percentiles: 25%−75%). Correlations between expression levels of target genes/proteins and muscle fibre phenotypic characteristics were explored using the Spearman correlation coefficient (r_s_); 95% confidence intervals were calculated. Correlation analysis was based on certain findings and therefore was not pre-planned. All statistical tests were carried out using the software GraphPad Prism v10.1.0 (GraphPad Software, San Diego, USA). The level of statistical significance was set at p<0.05.

## Results

### Phenotypic changes in myofibre characteristics

Patient demographic and lung function characteristics at the outset of the study are shown in table 1. Atrophic and nonatrophic patients with COPD did not differ in terms of age and severity of airflow obstruction but differed in terms of body mass index and FFMI (table 1). Prior to PR, there were not any significant differences in muscle fibre type distribution between groups; however, the mean fibre CSA was significantly lower in atrophic (3872±258 μm^2^) compared to nonatrophic (4509±198 μm^2^) patients with COPD secondary to lower CSA for IIa and IIx fibres.

**TABLE 1:** Demographic and lung function characteristics of nonatrophic and atrophic patients with COPD

Characteristics	Patients with COPD	
	Nonatrophic	Atrophic
**Patients n**	19	10
**Age years**	67.10±1.89	63.00±2.13
**Weight kg**	77.35±3.09	62.94±2.29*
**BMI kg**·**m^−2^**	28.81±1.17	21.50±0.66*
**FFM index kg**·**m^−2^**	19.04±0.40	15.64±0.38*
**FEV_1_ L**	1.18±0.12	0.98±0.14
**FEV_1_ % predicted**	44.07±4.84	37.52±6.13
**FVC L**	2.84±0.19	2.49±0.17
**FVC % predicted**	80.55±5.04	72.86±5.94

Data are presented as mean±sem. BMI: body mass index; FFM: fat-free mass; FEV_1_: forced expiratory volume in 1 s; FVC: forced vital capacity. *: p<0.05 significance level between nonatrophic and atrophic patients with COPD.

**FIGURE 1:**
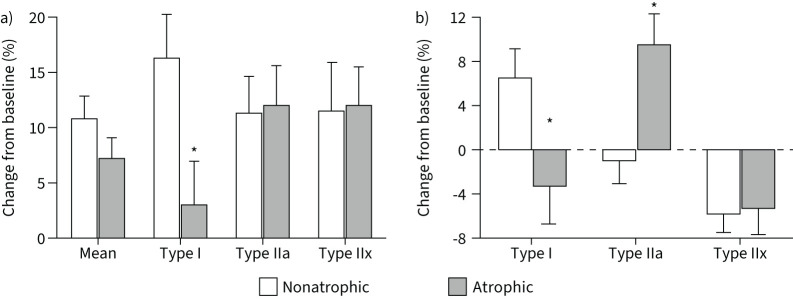
Effect of exercise-based pulmonary rehabilitation on vastus lateralis muscle fibre characteristics in nonatrophic and atrophic patients with COPD. Fractional changes in vastus lateralis muscle fibre morphological characteristics: a) cross-sectional area (µm^2^); b) fibre type distribution. Changes have been calculated from our previously published data [4]. *: differences (p<0.05) in atrophic compared to nonatrophic patients with COPD.

Figure 1 shows PR-induced changes in morphological characteristics of the vastus lateralis muscle in nonatrophic and atrophic patients with COPD. The fractional changes in vastus lateralis muscle fibre morphological characteristics depicted in figure 1 have been calculated from our previously published data [4]. PR significantly increased mean muscle fibre CSA in both COPD groups. In atrophic patients with COPD the increase in mean fibre CSA was mainly due to increased CSA in fibre type IIa and type IIx fibres (not fibre type I), whereas in nonatrophic patients with COPD the increase in mean fibre CSA was due to an increase in fibre type I and type IIx (figure 1). In response to PR, atrophic patients with COPD significantly increased the proportion of type IIa fibres, whereas nonatrophic patients with COPD increased the proportion of type I fibres. Post-PR, both groups exhibited a significant decrease in the distribution of type IIx fibres (figure 1).

### Expression of structural collagen proteins

**FIGURE 2:**
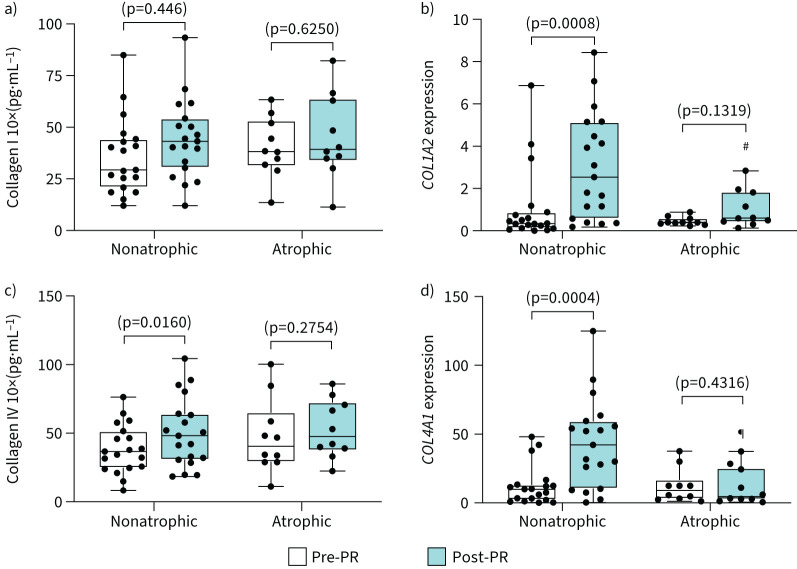
Collagen is increased only in nonatrophic patients with COPD post-pulmonary rehabilitation (PR). Comparisons between nonatrophic and atrophic patients with COPD pre- and post-PR. Boxplot graphs depict the median (black line) and lower and upper quartiles for: a) collagen type I protein levels and b) mRNA expression (*COL1A2*); c) collagen type IV protein levels and d) mRNA expression (*COL4A1*). The values for each individual participant are represented as black data points. Protein levels were measured with ELISA. mRNA expression was measured with quantitative real-time PCR; data are presented as fold changes relative to the housekeeping gene glyceraldehyde 3-phosphate dehydrogenase (*GAPDH*). Statistical significance within groups is shown on graphs and between groups is shown as: ^#^: p<0.05, ^¶^: p=0.005.

Post-PR collagen type I and type IV total protein and mRNA (COL1A2 and COL4A1, respectively) expression was significantly increased in nonatrophic patients with COPD (figure 2); no significant changes were observed in response to PR in mRNA (COL1A2 or COL4A1) or protein expression in atrophic patients with COPD (figure 2). Post-PR changes in COL1A1 mRNA expression were significantly greater in nonatrophic compared to atrophic patients with COPD (supplementary figure S1a).

### Expression of myogenic proteoglycans

**FIGURE 3:**
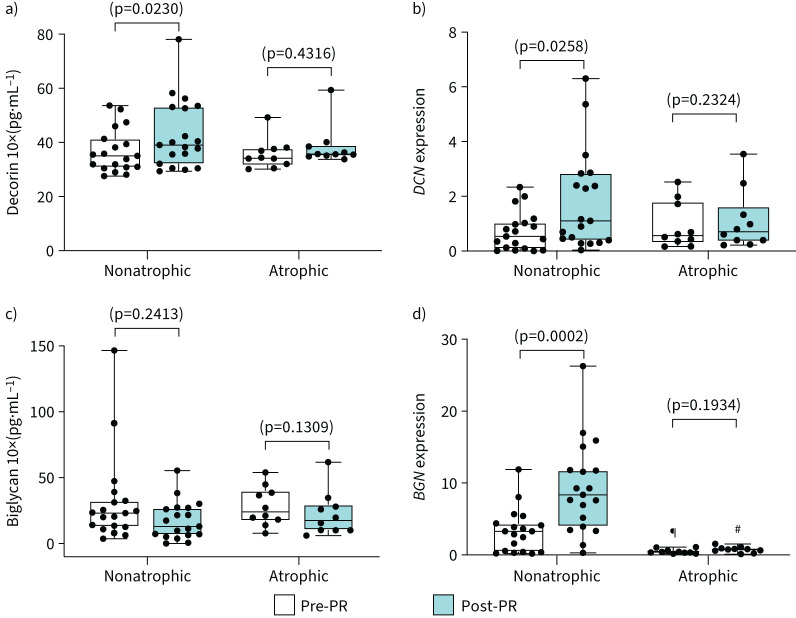
Expression of proteoglycans DCN and BGN is blunted in atrophic patients with COPD post-pulmonary rehabilitation (PR). Comparisons between nonatrophic and atrophic patients with COPD pre- and post-PR. Boxplot graphs depict the median (black line) and lower and upper quartiles for: a) decorin protein levels and b) mRNA expression (*DCN*); c) biglycan protein levels and d) mRNA expression (*BGN*). The values for each individual participant are represented as black data points. Protein levels were measured with ELISA. mRNA expression was measured with quantitative real-time PCR; data are presented as fold changes relative to the housekeeping gene glyceraldehyde 3-phosphate dehydrogenase (*GAPDH*). Statistical significance within groups is shown on graphs and between groups is shown as: ^#^: p<0.0001; ^¶^: p=0.007.

PR significantly increased decorin (*DCN*) at both mRNA and protein levels only in nonatrophic patients with COPD (figure 3a and 3b). There was no change in decorin at the mRNA or protein level in atrophic patients with COPD (figure 3a and 3b). Furthermore, PR induced a significant increase in biglygan (*BGN*) mRNA expression only in nonatrophic patients with COPD (figure 3d). Post-PR changes in biglygan mRNA expression were significantly greater in nonatrophic compared to atrophic patients with COPD (supplementary figure S1b).

### Expression of dystrophic and myogenic matricellular proteins

**FIGURE 4:**
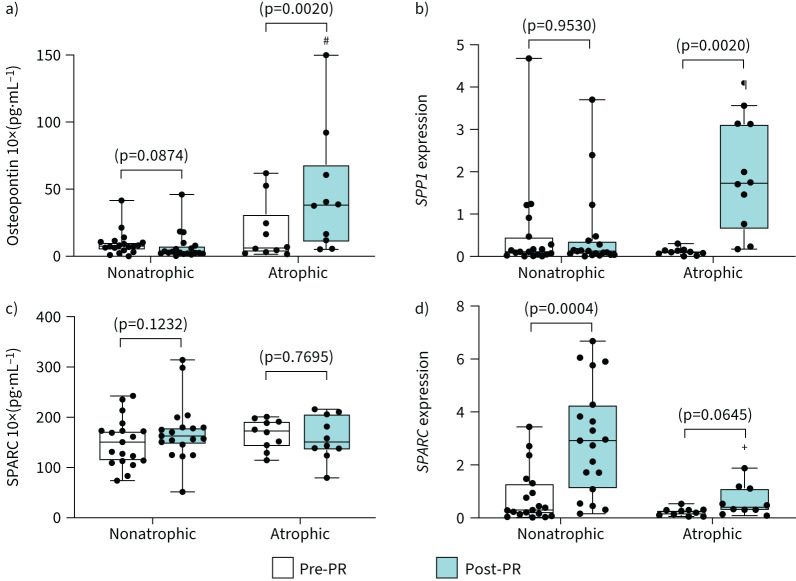
Matricellular proteins opposing levels of expression post-pulmonary rehabilitation (PR) in nonatrophic and atrophic patients with COPD. Comparisons between nonatrophic and atrophic patients with COPD pre- and post-PR. Boxplot graphs depict the median (black line) and lower and upper quartiles for: a) osteopontin protein levels and b) mRNA expression (*SPP1*); c) SPARC protein levels and d) mRNA expression (*SPARC*). The values for each individual participant are represented as black data points. Protein levels were measured with ELISA. mRNA expression was measured with quantitative real-time PCR; data are presented as fold changes relative to the housekeeping gene glyceraldehyde 3-phosphate dehydrogenase (*GAPDH*). Statistical significance within groups is shown on graphs and between groups is shown as: ^#^: p=0.0004; ^¶^: p=0.0009, ^+^: p=0.0014.

PR induced a significant increase in osteopontin at both the mRNA (*SPP1*) and protein level only in atrophic patients with COPD (figure 4a and 4b). Post-PR, the change in osteopontin (*SPP1*) expression was significantly greater in atrophic compared to nonatrophic patients with COPD (supplementary figure S1c). To appreciate whether there is a relationship between post-PR phenotypic changes and changes in the mRNA expression, we examined the association between changes in *SPP1* mRNA expression and myofibre phenotypic PR-induced adaptations. In atrophic patients with COPD, the increase from baseline in *SPP1* mRNA expression was negatively associated with the post-PR change in CSA for fibre type I CSA (r_s_ = −0.67, p=0.033). At protein level, post-PR levels from all COPD patients were positively correlated with the baseline levels (pre-PR) of osteopontin (r_s_ = −0.82, p=0.0001) (supplementary table S1).

SPARC levels did not change in response to PR in any of the COPD groups, despite a significant increase in *SPARC* mRNA expression in nonatrophic COPD (figure 4c and 4d). The post-PR change in *SPARC* mRNA expression was significantly greater in nonatrophic compared to atrophic patients with COPD (supplementary figure S1d), and these changes were negatively associated with type II CSA (r_s_ = −0.68, p=0.030) (supplementary table S1).

### Regulation of adhesive/de-adhesive glycoproteins

**FIGURE 5:**
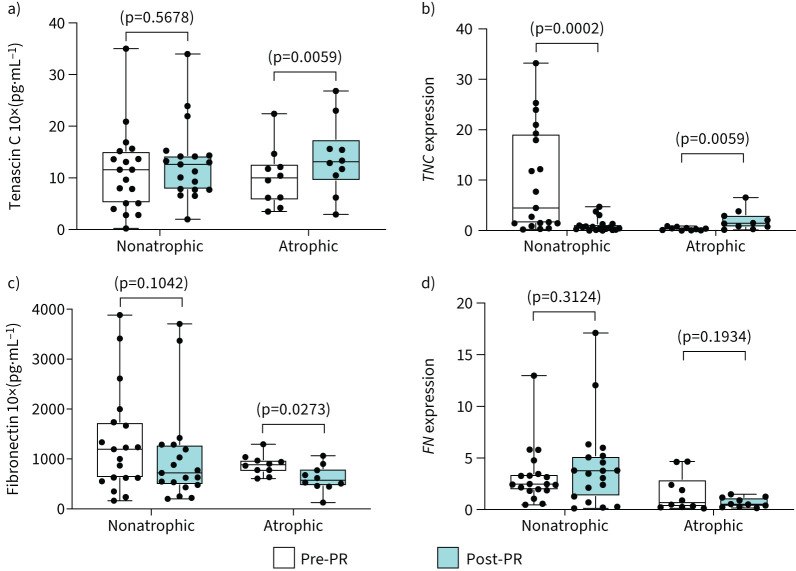
Atrophic patients with COPD upregulate tenascin C and downregulate fibronectin post-pulmonary rehabilitation (PR). Comparisons between nonatrophic and atrophic patients with COPD pre- and post-PR. Boxplot graphs depict the median (black line) and lower and upper quartiles for: a) tenascin C protein levels and b) mRNA expression (*TNC*); c) fibronectin protein levels and d) mRNA expression (*FN*). The values for each individual participant are represented as black data points. Protein levels were measured with ELISA. mRNA expression was measured with quantitative real-time PCR; data are presented as fold changes relative to the housekeeping gene glyceraldehyde 3-phosphate dehydrogenase (*GAPDH*).

PR induced significant increases in de-adhesive tenascin C protein and mRNA (*TNC*) levels only in atrophic patients with COPD (figure 5a and 5b). In nonatrophic patients with COPD, *TNC* mRNA expression was decreased post-PR (figure 5b) and was negatively associated with the post-PR increase in muscle fibre type I distribution (r_s_ = −0.83, p=0.008) and with type I CSA (r_s_ = −0.71, p=0.007) (supplementary table S1). Importantly, the adhesive fibronectin was significantly reduced in atrophic COPD post-PR at the protein level, albeit not at mRNA level (*FN*) (figure 5c and 5d). No significant PR-induced changes in fibronectin protein or mRNA levels were observed in nonatrophic patients with COPD (figure 5c and 5d). Post-PR, the protein level representing the ratio of fibronectin to osteopontin was significantly lower in atrophic compared to nonatrophic patients with COPD (supplementary figure S2).

**FIGURE 6:**
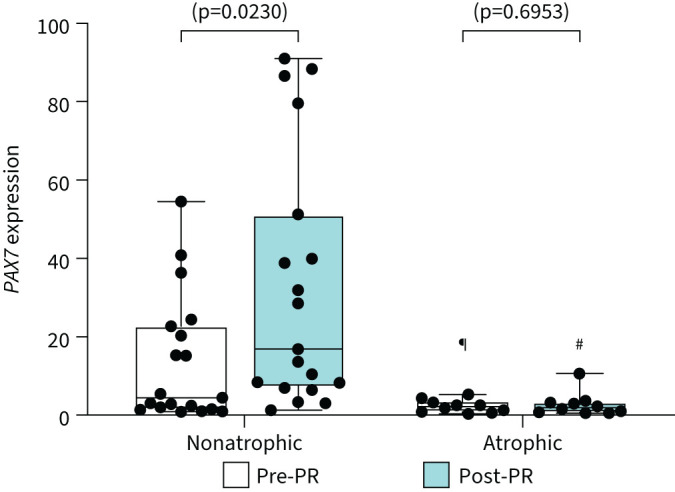
*PAX7* expression remains significantly lower in atrophic patients with COPD pre- and post-pulmonary rehabilitation (PR). Comparisons between nonatrophic and atrophic patients with COPD pre- and post-PR. Boxplot graphs depict the median (black line) and lower and upper quartiles for the mRNA expression of the paired box 7 gene (*PAX7*) mRNA expression. The values for each individual participant are represented as black data points. mRNA expression measured with quantitative real-time PCR; data are presented as fold changes relative to the housekeeping gene glyceraldehyde 3-phosphate dehydrogenase (*GAPDH*). Statistical significance within groups is shown on graphs and between groups is shown as: ^#^: p<0.0001; ^¶^: p=0.0445.

Considering that lower availability of fibronectin is responsible for compromised cell adherence and anchorage programmed cell death (anoikis) of satellite cells, the expression of satellite cells molecular marker (*PAX7*) is reported here [18, 19]. The mRNA expression of this unique biomarker for satellite cells (*PAX7*) was significantly lower prior to PR in atrophic compared to nonatrophic patients with COPD (figure 6). PR significantly increased *PAX7* mRNA expression only in nonatrophic patients with COPD (figure 6).

## Discussion

In response to an exercise-based PR programme the type of muscle fibre hypertrophic and/or phenotypic adaptation differs between atrophic and nonatrophic patients with COPD [11]. This study is the first to demonstrate that exercise-based PR induces diverse intramuscular ECM adaptations in atrophic compared to nonatrophic patients with COPD. Accordingly, mRNA and protein levels of expression of ECM biomarkers (collagen type I and IV and decorin) were significantly increased following PR only in nonatrophic patients with COPD. Conversely, post-PR, osteopontin, a protein known for its dystrophic effects, and tenascin C were upregulated at both mRNA and protein levels only in atrophic COPD, whereas fibronectin protein levels were decreased. These findings suggest that the attenuated PR-induced myofibre adaptation in response to PR in atrophic patients with COPD could be associated with impaired intramuscular ECM expression.

Our group recently showed that at baseline, gene expression of ECM molecules is significantly lower in atrophic compared to nonatrophic patients with COPD for collagen type I, fibronectin, tenascin C, biglycan and PAX7 [11]. Furthermore, it is known that altered ECM composition compromises the capacity for muscle adaptation [6]. In the present study, we investigated whether the attenuated training-induced myofibre adaptations in atrophic compared to nonatrophic patients with COPD [4] could be explained by a different pattern of adaptation in the ECM composition in response to exercise-based PR.

Osteopontin is a matricellular protein that does not subserve structural roles but operates as a modulator of cell-matrix interaction by binding to other ECM molecules, growth factors and cell-surface receptors [16, 20]. In older individuals, osteopontin is the most abundant senescence-associated secreted protein (SASP) and becomes elevated in young healthy individuals after muscle injury [21, 22]. Neutralisation of osteopontin improves muscle regeneration and induces upregulation of myogenic markers after injury in a murine model [23]. In Duchenne muscular dystrophy, osteopontin is implicated in disease severity and fibrosis [24]. Although there is credible evidence on the adverse impact of overexpression of osteopontin on muscle regeneration [23], while hypoxia and inflammation could also trigger its expression [25], this is the first study describing the involvement of osteopontin in limiting myofibre adaptation in atrophic patients with COPD. The profound PR-induced increase in osteopontin protein levels in atrophic COPD (figure 4) highlights the possible role of this matricellular protein in limiting myofibre growth following a programme of exercise training. Osteopontin may be a critical ECM component for the successful myofibre adaptation in patients with COPD. In support of this notion, baseline protein levels of osteopontin may constitute a predictive biomarker of PR phenotypic response in patients with COPD, due to the high correlation between the pre-PR and post-PR protein osteopontin levels. Given the role of osteopontin in muscle dystrophy [24], we observed an inverse association of osteopontin (*SPP1*) mRNA expression and the limited fibre type I hypertrophy in atrophic patients with COPD, thereby suggesting that OPN may be involved in adaptative resistance to type I fibre hypertrophy reported in these patients (figure 1). Other matricellular proteins, such as SPARC [26], have been described to have myokine function-inducing exercise-like adaptations. In our settings, however, SPARC mRNA expression was increased only in nonatrophic patients with COPD, and it was not positively associated with changes in PR-induced muscle hypertrophy in patients with COPD.

Mechanical loading induces hypertrophic growth by a discrete process of muscular repair in response to microtrauma and increased functional workload, evoking activation of muscle-cell precursors and ECM proteostasis [27, 28]. Fibronectin provides adherence to muscle-cell precursors, a mechanism essential for cell survival, differentiation and hypertrophic myonuclear accretion [18, 19, 27, 29]. It is conceivable that the PR-induced reduction in fibronectin (>six-fold) in atrophic patients with COPD (figure 5) may have compromised cell adhesion, possibly leading to satellite cell ECM-anchorage-dependent apoptosis (anoikis) [18, 19]. Although satellite cell density is similar among COPD patients with variable muscle mass [30], senescent satellite cells are predominant in atrophic patients with COPD, thereby suggesting the occurrence of repetitive episodes of unsuccessful replication and exhaustion of the replicative potential [30]. In support of this notion, we observed in post-PR that the expression of satellite cells’ unique molecular biomarker *PAX7* expression was increased only in nonatrophic patients with COPD (figure 6), and this increase was associated with an increase in fibre type I distribution. Therefore, our data suggest that PR may induce satellite cell renewal in nonatrophic COPD alongside cell differentiation through myogenic lineage leading to an increase in fibre type I distribution [31]. Conversely, in atrophic patients with COPD, the ECM micro-environment does not favour the maintenance of satellite cell function, limiting progression through the myogenic lineage and muscle regeneration [18, 19].

Furthermore, we found that the ratio of fibronectin/osteopontin was 16-fold lower in atrophic compared to nonatrophic patients with COPD post-PR (supplementary figure S2). Further reduction in fibronectin availability would be expected by covalent cross-linking with osteopontin *via* a molecular mechanism which firmly incorporates osteopontin into the ECM micro-environment [32]. The balance between adherence and de-adherence could be further constrained in atrophic patients with COPD, as fibronectin adhesive properties can be neutralised by the interaction of increased tenascin C protein levels post-PR in atrophic patients with COPD (figure 5) [33].

The measurement of tenascin C can reliably quantify connective tissue damage, as it is the major protein released following mechanical stress damage of necroptotic myofibres [33, 34]. In atrophic patients with COPD, increased levels of tenascin C protein post-PR indicate tissue stress damage and possibly myofibre necroptosis in response to repeated episodes of mechanical loading [33]. Further experiments supporting this hypothesis could be gained from investigating the necrosome signalling pathways and the primary executors of necroptosis (MLKL) [35]. Tenascin C acts as a necroptosis compensatory factor facilitating muscle regeneration by directly activating epidermal growth factor (EGF) signalling pathways on satellite cells [33, 34]. In patients with COPD, activation of EGF is negatively associated with the proportion of muscle fibres type I [36]. Taken together, our data suggest that the increased levels of tenascin C in atrophic patients with COPD would in turn enhance the activation of EGF, possibly leading to reduced type I fibre distribution post-PR (figure 1), and commensurate increase in type II fibre distribution [1]. Likewise, a study in mice demonstrated that tenascin C protects fast muscle fibres from deleterious consequences of mechanically induced microdamage [37]. In agreement, we demonstrated that decreased *TNC* mRNA expression in nonatrophic patients with COPD (figure 5) was associated with increased type I fibre distribution and hypertrophy. This finding indicates that TNC is associated with fibre type redistribution in patients with COPD. Collectively, we reason that the TNC orchestrates the molecular pathways integrating muscle repair into the load-dependent control of the striated muscle phenotype.

Important differences were also observed at the level of proteoglycans decorin and biglycan (figure 3), which share high homology and overlapping functions [38]. In addition to the protective role of stabilising muscle structure, decorin regulates muscle hypertrophic response by modulating the bioavailability of growth factors and by directly binding to and inactivating myostatin [39, 40]. Decorin hypertrophic activity increases the expression of myogenic factors MyoD1 and follistatin while reducing ubiquitin ligase atrogin1 and MuRF1 [39, 40]. Similarly to young healthy individuals [40], decorin was upregulated at the protein level in nonatrophic patients with COPD post-PR (figure 3), possibly implicating this ECM in the hypertrophic response to exercise training [39, 40]. Considering that decorin is the most abundant proteoglycan capable of regulating myogenesis by acting as a direct antagonist of myostatin and repressing transforming growth factor-β signalling [39], it was not a surprise to observe no changes in decorin at the mRNA or protein level in atrophic patients with COPD (figure 3). This finding may justify previously reported unaltered levels of myostatin, and increased atrogin1 and MurF1 protein levels post-PR in atrophic patients with COPD [4].

Collagen is the most abundant ECM in muscle. Changes in muscle collagen type I, including increased protein and mRNA expression levels on both *COL1A1* and *COL1A2* chains, indicate PR-induced structural adaptations [6, 9]. Although atrophic patients with COPD had increased exercise capacity, no significant changes in collagen type I and structural adaptation were observed. Additionally, PR did not induce changes in collagen type IV in atrophic patients with COPD, which is a protein that forms a scaffolding network at the basement membrane, integrating the ECM micro-environment and cell signalling [6, 9].

While we recognise upregulation of ECMs with dystrophic effects, participation in the exercise-based PR programme could overall be considered beneficial for atrophic patients with COPD in terms of inducing significant muscle fibre morphological changes (figure 1). While aerobic training improves exercise tolerance and symptom perception, muscle strengthening exercise to increase muscle mass should constitute a highly significant component of the exercise programme in atrophic patients with COPD [1].

Future research studies should investigate whether the effects of exercise training on muscle fibre morphological and protein expression levels translate into functional muscle adaptations such as improved quadriceps muscle strength and endurance in atrophic patients with COPD. Furthermore, future studies should identify the optimal modality of exercise training (with emphasis on resistance or endurance training) in terms of improving functional outcomes in this COPD patient cohort.

The present study is not without limitations. Participants in this study were all male. Hence, the findings of this study are limited to only representing male patients and cannot be generalised to the whole COPD population. Furthermore, due to the retrospective nature of the study, we acknowledge a possible lack of power for this body of work appreciating that the sample size is generally low, making the calculated magnitude of correlation analysis unstable. While the list of biomarkers studied is not exhaustive, they can be related to our earlier study describing ECM baseline conditions in atrophic and nonatrophic patients with COPD [11]. Further research is needed to determine whether modifying ECM signalling response would positively impact muscle mass growth. Consistent with previous studies [9, 11], our findings reinforce the notion that skeletal muscle relies on a well-coordinated ECM response to maintain its homeostasis and regenerative capacity.

In conclusion, exercise-based PR leads to diverse structural and myogenic ECM adaptative responses in atrophic compared to nonatrophic patients with COPD. These responses may play a role in limiting the extent and influencing the pattern of myofibre adaptation in atrophic patients with COPD in response to PR.

## Supplementary material

10.1183/23120541.00543-2024.Supp1 **Please note:** supplementary material is not edited by the Editorial Office, and is uploaded as it has been supplied by the author.
Supplementary material 00543-2024.SUPPLEMENT
Figure S1 00543-2024.FIGURES1
Figure S2 00543-2024.FIGURES2
