# Supplementary material for: Role of pulmonary rehabilitation in extracellular matrix protein expression in vastus lateralis muscle in atrophic and nonatrophic patients with COPD
Source: ERJ Open Res. 2025 Jan 20;11(1):00543-2024. doi: 10.1183/23120541.00543-2024 (PMC11745040; doi:10.1183/23120541.00543-2024)
Supplement: Supplementary file 1 [file 00543-2024.SUPPLEMENT.pdf]

Supplementary Table 1: Spearman correlation with 95% confidence intervals between mRNA expression of ECMs and muscle fibre morphological changes (Δ) in non-atrophic and atrophic COPD

	Non-atrophic COPD				Atrophic COPD			
	Δ Fibre type		Δ CSA		Δ Fiber type		Δ CSA	
mRNA	Type I	Type II	Type I	Type II	Type I	Type II	Type I	Type II
ΔCOL1A1	-0.188	-0.021	0.140	0.121	0.626	-0.647	0.646	0.378
ΔCOL4A1	-0.474	0.344	0.020	-0.123	-0.753	0.699	0.574	0.102
ΔCOL1A2	-0.040	0.426	-0.039	0.172	-0.459	0.400	0.980	0.772
ΔFN	0.045	-0.199	-0.403	0.044	0.466	-0.417	0.709	0.216
ΔTNC	-0.833	0.708	-0.713	-0.009	0.350	-0.370	0.135	0.795
ΔSPARC	0.266	0.328	-0.512	-0.681	0.370	-0.018	0.622	0.400
ΔSPP1	-0.176	-0.034	-0.252	-0.154	0.077	0.164	-0.674	0.094
ΔDCN	-0.504	0.496	-0.250	0.078	-0.607	0.383	0.388	0.124
ΔBGN	-0.011	0.351	-0.084	-0.634	0.093	-0.028	0.569	0.097
