# Supplementary material for: Role of pulmonary rehabilitation in extracellular matrix protein expression in vastus lateralis muscle in atrophic and nonatrophic patients with COPD
Source: ERJ Open Res. 2025 Jan 20;11(1):00543-2024. doi: 10.1183/23120541.00543-2024 (PMC11745040; doi:10.1183/23120541.00543-2024)
Supplement: Supplementary file 2 [file 00543-2024.FIGURES1.pdf]

Figure S1

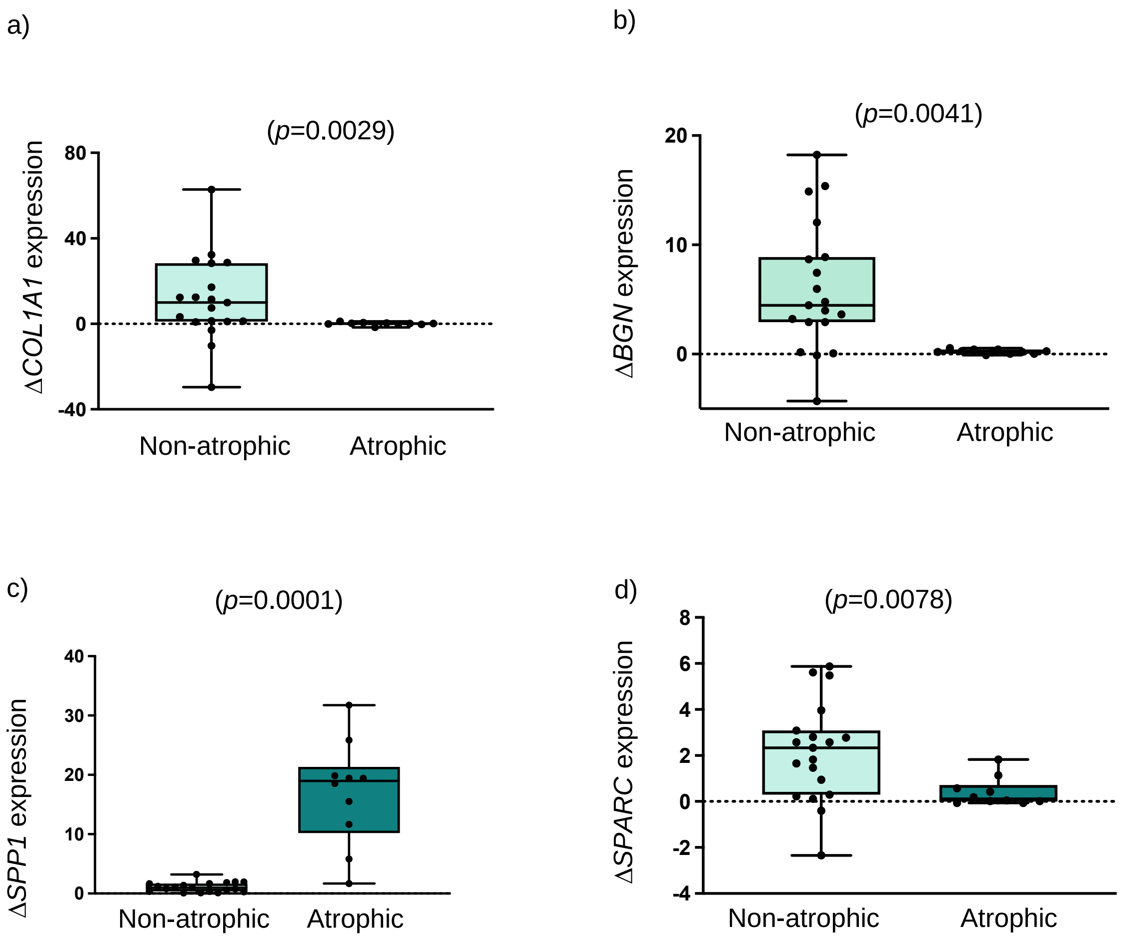

Figure S1: Osteopontin is highly upregulated in atrophic patients with COPD post-PR.
Atrophic and non-atrophic patients with COPD patients were compared in terms of the magnitude of changes (Δ= post-PR – pre-PR) for mRNA expression of collagen type I (*COL1A1*) (a), biglycan (*BGN*) (b), osteopontin (*SPP1*) (c), and *SPARC (d)*. Data are presented as boxplots showing the median (black line) and lower and upper quartiles. Individual participant values are represented as filled data points. The qRT-PCR data are presented as fold changes relative to the housekeeping gene glyceraldehyde 3-phosphate dehydrogenase (*GAPDH*).
