# Supplementary material for: Role of pulmonary rehabilitation in extracellular matrix protein expression in vastus lateralis muscle in atrophic and nonatrophic patients with COPD
Source: ERJ Open Res. 2025 Jan 20;11(1):00543-2024. doi: 10.1183/23120541.00543-2024 (PMC11745040; doi:10.1183/23120541.00543-2024)
Supplement: Supplementary file 3 [file 00543-2024.FIGURES2.pdf]

Figure S2

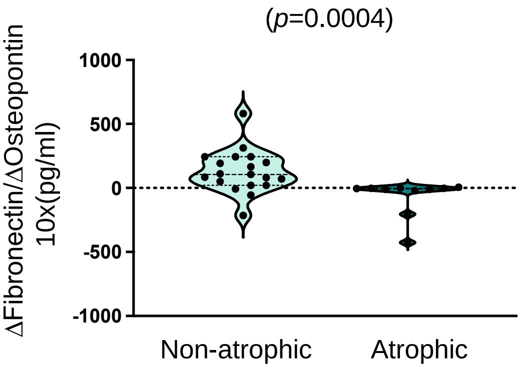

Figure S2: In atrophic patients with COPD the ratio fibronectin/osteopontin does not change post-pulmonary PR.

Atrophic and non-atrophic patients with COPD patients were compared relative to the magnitude of changes (Δ= post-PR – pre-PR) of mRNA expression relative to the ratio of fibronectin to osteopontin. Data are presented as violin plot showing the median (black line) and lower and upper quartiles. Individual participant values are represented as black data points. Protein abundance was quantified using ELISA.
